# Cardiovascular Disease Risk Estimation Using Blood Biomarkers and the QRISK3 Stratification Tool: A Cross‐Sectional Study in Jordan

**DOI:** 10.1002/hsr2.72388

**Published:** 2026-06-04

**Authors:** Ahmed Salem, Fanar Alsmarat, Tasneem Al‐Awaideh, Layth Al‐Ramahi, Yousef Shawawrah

**Affiliations:** ^1^ Department of Anatomy, Physiology and Biochemistry, Faculty of Medicine The Hashemite University Zarqa Jordan; ^2^ Faculty of Applied Medical Science The Hashemite University Zarqa Jordan; ^3^ Medical Student The Hashemite University Zarqa Jordan

**Keywords:** cardiovascular diseases, Jordanian population, passive smoking, QRISK3, smoking

## Abstract

**Background and Aims:**

Cardiovascular diseases (CVD) are the leading cause of death in Jordan. This study aimed to estimate the prevalence of 10‐year CVD risk as predicted by the QRISK3 tool and assess fibrinogen levels and lipid profile parameters as additional indicators of CVD risk in a Jordanian sample.

**Methods:**

This cross‐sectional study was conducted in Amman, Jordan. Eligible participants aged 25–64 years provided informed consent and completed a baseline questionnaire. Blood samples were collected for fibrinogen and lipid profile analyses. This study followed the STROBE guidelines for reporting observational studies.

**Results:**

A total of 251 participants were recruited for this study. Among them, 78 (31.1%) had ≥ 10% QRISK3 score, and 36 (14.3%) had > 20% QRISK3 score. Active smokers had a higher prevalence of both ≥ 10% and > 20% QRISK3 scores compared to secondhand and never‐smokers (*p* < 0.001 and *p* = 0.038, respectively). The prevalence of atrial fibrillation was higher in active smokers (20%) and secondhand smokers (25.4%) compared to never‐smokers (6.3%) (*p* = 0.014). A total of 195 participants (77.7%) exhibited at least one non‐optimal lipid profile level. Smokers of more than 10 years showed a significantly higher prevalence of low HDL levels compared to those who had smoked for fewer years (*p* = 0.034). Median fibrinogen levels were similar across smoking statuses (active smokers: 268 mg/dL, never‐smokers: 275 mg/dL, and secondhand smokers: 258 mg/dL, *p* = 0.456).

**Conclusion:**

While the findings of this study cannot be generalized to the entire Jordanian population due to convenience sampling and the limited sample size, they highlight that a substantial proportion of participants are at elevated CVD risk. This underscores the importance of incorporating routine CVD risk screening into primary healthcare to identify high‐risk individuals and enable early preventive interventions.

## 1 Introduction

Cardiovascular diseases (CVD) are the leading cause of death in Jordan, accounting for more than one‐quarter of all deaths, despite the country's predominantly young population [1, 2, 3]. Over the past three decades, CVD‐related mortality has increased nearly threefold, rising from 4534 deaths in 1990 to 11,798 deaths in 2019 [3]. Around three‐quarters of Jordanian adults have at least two modifiable CVD risk factors, such as smoking, obesity, and dyslipidemia [4].

Smoking is one of the most significant modifiable CVD risk factors, increasing risk for both active and secondhand smokers compared with never smokers [5, 6, 7]. Jordan has one of the highest rates of smoking worldwide, with over 41% of the population actively smoking and more than 80% exposed to secondhand smoke [8]. One of the key mechanisms linking smoking to CVD is its effect on fibrinogen, an acute‐phase glycoprotein primarily involved in the blood coagulation process; active smokers exhibit 60% higher fibrinogen levels than both secondhand and never smokers, with secondhand smokers also showing elevated levels compared to never smokers [9]. Elevated fibrinogen levels are associated with 15% increase in CVD risk [10].

Dyslipidemia is another major modifiable contributor to CVD in Jordan [11]. A 2019 national cross‐sectional study revealed that 81.6% of Jordanian adults had dyslipidemia, yet only 9.3% were aware of their condition, underscoring a significant gap in diagnosis and awareness [12].

The high prevalence of smoking and other CVD risk factors, including dyslipidemia, combined with the substantial gap between prevalence and awareness, indicates that CVD will remain a major public health concern in Jordan, and underscores the need for comprehensive risk assessment and management strategies tailored to the Jordanian population. Risk stratification is one of the most effective approaches for CVD prevention. Currently, multiple tools exist for estimating CVD risk, including the QRISK3 calculator, a well‐validated risk assessment tool known for its accuracy and reliability in predicting 10‐year CVD risk [13]. It is recommended by the National Institute for Health and Care Excellence (NICE) for routine CVD risk assessment [14]. The QRISK3 tool incorporates a wide range of demographic and clinical variables, including height, weight, smoking status, relevant medical and family history, systolic blood pressure, and its standard deviation from the two most recent readings, as well as the total cholesterol to high‐density lipoprotein (HDL) ratio obtained from a non‐fasting blood sample [13]. However, its applicability to the Jordanian population remains relatively unexplored.

This study aimed to estimate the prevalence of 10‐year CVD risk as predicted by the QRISK3 tool and to assess fibrinogen levels and lipid profile parameters as additional indicators of CVD risk in the Jordanian population.

## 2 Methods

### 2.1 Study Design and Population

This cross‐sectional study was conducted in Amman, Jordan, between 14 November 2023 and 2 March 2024. Ethical approval was obtained from the Institutional Review Board of the Hashemite University (Approval No. 33/5/2022/2023), and written informed consent was obtained from all participants prior to enrollment.

Participants were recruited using convenience sampling from Amman and Madaba, Jordan, by trained research assistants and medical students from the Hashemite University. No formal sample size calculation was performed due to budgetary constraints; instead, all eligible participants available during the study period were included to achieve the largest feasible sample size. The recruitment process aimed to approximate the demographics of the Jordanian adult population in terms of age and gender. To achieve this, the recruitment team actively monitored enrollment numbers throughout the study period and adjusted recruitment efforts to target underrepresented groups accordingly.

Eligibility was assessed using a standardized questionnaire administered prior to enrollment (Supplementary File 1). Participants were required to be Jordanians, aged 25–64 years, and free from chronic illnesses, specifically cancer and CVD.

### 2.2 Smoking Status Classification

Smoking status was assessed according to definitions from the National Center for Health Statistics, with additional criteria introduced to avoid overlap between categories:

- Active smokers (everyday smokers) were defined as adults who have smoked at least 100 cigarettes in their lifetime and currently smoke daily.
- Never‐smokers were defined as adults who have never smoked or have smoked fewer than 100 cigarettes in their lifetime. To avoid confusing never‐smokers with secondhand smokers, we added the criterion that never‐smokers are not regularly exposed to environmental tobacco smoke at home or workplaces.
- Secondhand smokers were defined as adults who have never smoked or have smoked fewer than 100 cigarettes in their lifetime but are regularly exposed to environmental tobacco smoke. Exposure was defined as living with a smoker or working in an environment where smoking is permitted or regularly occurs indoors, with exposure of at least 6 h per day. This criterion was added to specifically ensure and highlight meaningful passive smoking exposure in this group.

Carbon monoxide (CO) monitoring was performed for quality assurance of smoking status classification, but it was not used as an inclusion or exclusion criterion.

### 2.3 QRISK3 Data Collection and Risk Categorization

The QRISK3 calculator estimates an individual's 10‐year risk of CVD using a Cox proportional hazards model [13]. It incorporates demographic, clinical, and lifestyle variables, including age, gender, ethnicity, smoking status, diabetes, family history of CVD, chronic kidney disease, atrial fibrillation, blood pressure treatment, migraine, rheumatoid arthritis, systemic lupus erythematosus, severe mental illness, use of atypical antipsychotics or oral steroids, erectile dysfunction, cholesterol/HDL ratio, systolic blood pressure, blood pressure variability, and body mass index (BMI).

In our study, all required QRISK3 variables were collected through a standardized questionnaire, except for two:

1. The question regarding diagnosis or treatment of erectile dysfunction was excluded to minimize response bias due to cultural sensitivities.
2. The optional question regarding the standard deviation of systolic blood pressure readings was omitted to reduce potential recall bias from participant‐reported measurements.

Objective measurements, including height, weight, CO levels, and systolic blood pressure, were obtained directly by the trained research assistants and medical students.

Participants were categorized as low risk (<10%), moderate‐to‐high risk (≥ 10%), or high risk (> 20%) based on their QRISK3 scores.

### 2.4 Blood Sampling

Blood samples (5 mL) were collected from each participant. Fasting was not required prior to blood collection. From each sample, 1 mL was transferred into a 3.2% buffered sodium citrate tube (blue‐top; Samplix®, Kremsmünster, Austria) for fibrinogen measurement, while 2 mL was transferred into a plain tube (Samplix®, Kremsmünster, Austria) for lipid profile assessment. The plain tube was allowed to clot for 5–10 min before processing. Both tubes were centrifuged at 3600 rpm for 15 min at room temperature. Plasma and serum samples were properly labeled and stored at –20°C until analysis. All laboratory analyses were performed at Mega Lab Medical Laboratory according to manufacturer protocols.

### 2.5 Lipid Profile Analysis

Serum concentrations of total cholesterol, triglycerides, HDL, and low‐density lipoprotein cholesterol (LDL) were measured using homogeneous enzymatic colorimetric tests on a fully automated Roche/Hitachi Cobas c system (Roche/Basel). All samples were analyzed following the manufacturer's protocols.

### 2.6 Fibrinogen Measurement

Fibrinogen levels in sodium‐citrate plasma were measured using automated coagulation analyzers (Sysmex) following the Clauss method, with the Dade® Thrombin Reagent (Siemens Healthcare Diagnostics Products GmbH). All samples were analyzed following the manufacturer's protocols.

### 2.7 Statistical Analysis

Statistical analyses were performed using the Statistical Package for Social Sciences (SPSS) version 25 (IBM). Descriptive statistics were used to determine the prevalence of variables. The chi‐square test was used to assess the correlation between categorical variables. The Kruskal–Wallis test was used to compare more than 2 mean ranks according to smoking status. A *p*‐value of less than 0.05 was considered statistically significant.

## 3 Results

### 3.1 Demographic Characteristics of the Study Population

A total of 251 participants were enrolled in this study, including 125 active smokers (49.8%), 63 secondhand smokers (25.1%), and 63 never‐smokers (25.1%). Among active smokers, 30 (24%) were light smokers (≤10 cigarettes/day), 45 (36%) were moderate smokers (11–19 cigarettes/day), and 50 (40%) were heavy smokers (≥ 20 cigarettes/day).

The median age of the study population was 43 years (range: 25–64), with a median BMI of 29.0 kg/m² (range: 17.1–53.3) and a median CO level of 13.07 parts per million (ppm) (range: 1–51), Table 1.

**TABLE 1:** Demographic profile of participants and distribution of QRISK3 scores by smoking status.

Variable	All participants (*n* = 251)	Active smokers (*n* = 125)	Secondhand smokers (*n* = 63)	Never smokers (*n* = 63)	*p* value
Gender					*p* = 0.998
Males, *n* (%)	135 (53.8%)	67 (53.6%)	34 (53.9%)	34 (53.9%)	
Females, *n* (%)	116 (46.2%)	58 (46.4%)	29 (46.1%)	29 (46.1%)	
Age					*p* = 0.976
Median (range)	43 years (25–64)	43 years (25–64)	40 years (25–64)	44 years (25–64)	
25–54 years, *n* (%)	226 (90.0%)	110 (88.0%)	56 (88.9%)	56 (88.9%)	
55–64 years, *n* (%)	25 (10.0%)	15 (12.0%)	7 (11.1%)	7 (11.1%)	
BMI					*p* = 0.295
Median (range)	29.025 (17.1–53.3)	27.8 (17.1–48.4)	29.1 (17.4–48.0)	29.9 (18.7–53.3)	
Carbon Monoxide					*p* < 0.0001
Median (range)	13.07 (1–51)	18 (2–51)	5 (1–38)	3 (1–12)	
CO < 10 PPM	135 (53.8%)	27 (21.6%)	47 (74.6%)	62 (98.4%)	
CO ≥ 10 PPM	116 (46.2%)	98 (78.4%)	16 (25.4%)	1 (1.6%)	
≥ 10% QRISK3 Score					*p* < 0.0001
*n* (%)	78 (31.1%)	54 (43.2%)	14 (22.2%)	10 (15.9%)	
> 20% QRISK3 Score					*p* = 0.038
*n* (%)	36 (14.3%)	25 (20.0%)	6 (9.5%)	5 (7.9%)	

Abbreviations: BMI, body mass index; CO, carbon monoxide.

### 3.2 QRISK3‐Based Cardiovascular Disease Risk Assessment

The median QRISK3 score among all participants was 3.6% (range: 0.10%–80.0%). Active smokers had a significantly higher median QRISK3 score of 7.70% (range: 0.10%–80.0%) compared to secondhand smokers (median: 2.7%, range: 0.10%–46.5%) and never‐smokers (median: 2.3%, range: 0.10%–65.3%) (*p* = 0.002).

Overall, 78 participants (31.1%) had a ≥ 10% QRISK3 score (moderate to high risk). This proportion was highest among active smokers (43.2%), followed by secondhand smokers (22.2%) and never‐smokers (15.9%) (*p* < 0.0001). High CVD risk (QRISK3 score > 20%) was observed in 36 participants (14.3%), with the highest prevalence among active smokers (20.0%) compared to secondhand smokers (9.5%) and never‐smokers (7.9%) (*p* = 0.038), Table 1.

### 3.3 QRISK3 Risk Factors Distribution

The median cholesterol/HDL ratio among all participants was 4.5 (range: 2–9.8), and the median systolic blood pressure was 129 mmHg (range: 88–284 mmHg).

Chronic kidney disease, systemic lupus erythematosus, severe mental illness, and atypical antipsychotic use were not reported by any participants.

Most QRISK3 risk variables did not differ significantly across smoking groups, including hypertension treatment, family history of early angina or heart attack, migraines, rheumatoid arthritis, steroid use, and diabetes (all *p* > 0.05), Table 2.

**TABLE 2:** Association between smoking status and QRISK3 risk variables.

Risk variables	Smoking status	Yes *n* (%)	No *n* (%)	$x^{2}$	*p* value
Undergoing blood pressure treatment	Active smokers	16 (12.0%)	109 (87.2%)	0.286	0.867
	Never‐smokers	7 (11.1%)	56 (88.9%)		
	Secondhand smokers	9 (14.3%)	54 (85.7%)		
Had angina or a heart attack in a first‐degree relative under the age of 60	Active smokers	23 (18.4%)	102 (81.6%)	0.136	0.934
	Never‐smokers	13 (20.6%)	50 (79.4%)		
	Secondhand smokers	12 (19.0%)	51 (81.0%)		
Atrial fibrillation	Active smokers	25 (20%)	100 (80%)	8.493	**0.014**
	Never‐smokers	4 (6.3%)	59 (99.7%)		
	Secondhand smokers	16 (25.4%)	47 (74.6%)		
Migraines	Active smokers	15 (12%)	110 (88%)	4.288	0.117
	Never‐smokers	3 (4.8%)	60 (95.2%)		
	Secondhand smokers	3 (4.8%)	60 (95.2%)		
Rheumatoid arthritis	Active smokers	10 (8%)	115 (92%)	1.102	0.576
	Never‐smokers	3 (4.8%)	60 (95.2%)		
	Secondhand smokers	3 (4.8%)	60 (95.2%)		
Taking steroid tablets regularly	Active smokers	1 (0.8%)	124 (99.2%)	1.012	0.603
	Never‐smokers	0	63 (100%)		
	Secondhand smokers	0	63 (100%)		
Taking atypical antipsychotic medication	Active smokers	—	125 (100%)	—	—
	Never‐smokers	—	63 (100%)		
	Secondhand smokers	—	63 (100%)		
Chronic kidney disease (stage 3, 4, or 5)	Active smokers	—	125 (100%)	—	—
	Never‐smokers	—	63 (100%)		
	Secondhand smokers	—	63 (100%)		
Systemic lupus erythematosus	Active smokers	—	125 (100%)	—	—
	Never‐smokers	—	63 (100%)		
	Secondhand smokers	—	63 (100%)		
Severe mental illness	Active smokers	—	125 (100%)	—	—
	Never‐smokers	—	63 (100%)		
	Secondhand smokers	—	63 (100%)		
Diabetes	Active smokers	Type 1: 1 (0.8%) Type 2: 12 (9.6%)	112 (89.6%)	3.265	0.514
	Never‐smokers	Type 1: 2 (3.1%) Type 2: 4 (6.3%)	57 (90.5%)		
	Secondhand smokers	Type 1: 2 (3.1%) Type 2: 3 (4.8%)	58 (92%)		

*Note:* Bold value indicates statistically significant.

Atrial fibrillation was the only variable showing statistically significant differences across smoking groups, with higher prevalence among active smokers (20.0%) and secondhand smokers (25.4%) compared to never‐smokers (6.3%) (*p* = 0.014).

### 3.4 Lipid Profile and Dyslipidemia Prevalence

Among all participants, the median total cholesterol level was 184 mg/dL (range: 107–370 mg/dL), the median HDL level was 43 mg/dL (range: 22–94 mg/dL), the median LDL level was 118 mg/dL (range: 59–245 mg/dL), and the median triglyceride level was 156 mg/dL (range: 37–782 mg/dL). The median non‐HDL cholesterol level was 140 mg/dL (range: 71–237 mg/dL), the median LDL/HDL ratio was 2.9 (range: 0.9–7.1), and the median total cholesterol/HDL ratio was 4.5 (range: 2–9.8). No statistically significant differences were observed in any lipid profile parameter across smoking groups (all *p* > 0.05), Table 3.

**TABLE 3:** Median lipid profile levels among active smokers, never‐smokers, and secondhand smokers.

Lipid parameters	Smoking status	Median	Mean rank	Kruskal–Wallis	*p* value
HDL (mg/dL)	Active smokers	41	11.594	5.207	0.074
	Never‐smokers	45	140.17		
	Secondhand smokers	44	131.79		
LDL (mg/dL)	Active smokers	117	126.75	0.541	0.763
	Never‐smokers	120	129.90		
	Secondhand smokers	117	120.62		
Non‐HDL (mg/dL)	Active smokers	144	130.44	1.214	0.454
	Never‐smokers	140	125.05		
	Secondhand smokers	136	118.15		
LDL/HDL	Active smokers	3	133.37	2.600	0.273
	Never‐smokers	2.8	119.83		
	Secondhand smokers	2.7	117.55		
Total cholesterol/HDL ratio	Active smokers	4.5	134.38	3.325	0.190
	Never‐smokers	4.1	117.10		
	Secondhand smokers	4.1	118.28		
Total cholesterol (mg/dL)	Active smokers	185	127.67	0.554	0.758
	Never‐smokers	183	128.67		
	Secondhand smokers	184	120.16		
Triglyceride (mg/dL)	Active smokers	159	127.11	0.062	0.970
	Never‐smokers	146	124.51		
	Secondhand smokers	155	125.29		

Abbreviations: HDL, high‐density lipoprotein cholesterol; LDL, low‐density lipoprotein cholesterol.

Overall, 195 participants (77.7%) had at least one non‐optimal lipid profile value. When stratified by smoking status, the proportions of participants with non‐optimal lipid values did not differ significantly across smoking groups for any parameters (all *p* > 0.05), Table 4.

**TABLE 4:** Distribution of non‐optimal lipid profile values by smoking status.

Lipid profile parameter	Overall (*n* = 251)	Active smokers (*n* = 125)	Never smokers (*n* = 63)	Secondhand smokers (*n* = 63)	$x^{2}$	*p* value
HDL (mg/dL) < 35, *n* (%)	41 (16.3%)	25 (20.0%)	8 (12.7%)	8 (12.7%)	2.448	0.294
LDL (mg/dL) > 130, *n* (%)	84 (33.5%)	46 (36.8%)	22 (34.9%)	16 (25.4%)	2.526	0.283
Non‐HDL (mg/dL) > 130, *n* (%)	149 (59.4%)	74 (59.2%)	38 (60.3%)	37 (58.7%)	0.036	0.982
LDL/HDL > 3.6, *n* (%)	54 (21.5%)	31 (24.8%)	13 (20.6%)	10 (15.9%)	2.015	0.365
Total cholesterol/HDL ratio ≥ 3.8, *n* (%)	168 (66.9%)	89 (71.2%)	38 (60.3%)	41 (65.1%)	2.372	0.305
Total cholesterol (mg/dL) ≥ 200, *n* (%)	85 (33.9%)	47 (37.6%)	20 (31.7%)	18 (28.6%)	1.693	0.429
Triglyceride (mg/dL) ≥ 150, *n* (%)	132 (52.6%)	69 (55.2%)	30 (47.6%)	33 (52.4%)	0.967	0.617

Abbreviations: HDL, high‐density lipoprotein cholesterol; LDL, low‐density lipoprotein cholesterol.

### 3.5 Lipid Profile Stratified by Smoking Intensity and Duration

Among active smokers, the proportions of participants with non‐optimal lipid profile values did not differ significantly according to the number of cigarettes smoked per day (all *p* > 0.05). However, when stratified by smoking duration, participants who had smoked for more than 10 years exhibited a significantly higher prevalence of low HDL levels (<35 mg/dL) compared to those who had smoked for 10 years or less (25.9% vs. 9.1%, *p* = 0.034), Table 5.

**TABLE 5:** Proportion of smokers with non‐optimal lipid profiles by daily cigarette consumption and smoking duration.

Lipid profile parameter	Smoking intensities and duration	*N* (%)	Chi‐square	*p* value
HDL (mg/dL) < 35	1–10 cigarettes 11–20 cigarettes > 20 cigarettes	5 (16.7%) 8 (17.8%) 12 (24.0%)	0.847	0.655
	≤ 10 years > 10 years	4 (9.1%) 21 (25.9%)	5.051	**0.034**
LDL (mg/dL) > 130	1–10 cigarettes 11–20 cigarettes > 20 cigarettes	10 (33.3%) 19 (42.2%) 17 (34.0%)	0.892	0.640
	≤ 10 years > 10 years	17 (38.6%) 29 (35.8%)	0.098	0.754
Non‐HDL (mg/dL) > 130	1–10 cigarettes 11–20 cigarettes > 20 cigarettes	15 (50.0%) 28 (62.2%) 31 (62.0%)	1.384	0.501
	≤ 10 years > 10 years	27 (61.4%) 47 (58.0%)	0.132	0.717
LDL/HDL > 3.6	1–10 cigarettes 11–20 cigarettes > 20 cigarettes	7 (23.3%) 9 (20.0%) 15 (30.0%)	1.315	0.518
	≤ 10 years > 10 years	8 (18.2%) 23 (28.4%)	1.595	0.207
Total cholesterol/HDL ratio ≥ 3.8	1–10 cigarettes 11–20 cigarettes > 20 cigarettes	17 (56.7%) 34 (75.6%) 38 (76.0%)	4.068	0.131
	≤ 10 years > 10 years	30 (68.2%) 59 (72.8%)	0.302	0.583
Total cholesterol (mg/dL) ≥ 200	1–10 cigarettes 11–20 cigarettes > 20 cigarettes	10 (33.3%) 19 (42.2%) 18 (36.0%)	0.697	0.706
	≤ 10 years > 10 years	17 (38.6%) 30 (37.0%)	0.031	0.860
Triglyceride (mg/dL) ≥ 150	1–10 cigarettes 11–20 cigarettes > 20 cigarettes	15 (50.0%) 22 (48.9%) 32 (64.0%)	2.619	0.270
	≤ 10 years > 10 years	21 (47.7%) 48 (59.3%)	1.533	0.216

*Note:* Bold value indicates statistically significant.

Abbreviations: HDL, high‐density lipoprotein cholesterol; LDL, low‐density lipoprotein cholesterol.

### 3.6 Fibrinogen Levels

The median fibrinogen level for the study population was 268 mg/dL (range: 180–539 mg/dL). Median fibrinogen levels by smoking status were 268.0 mg/dL among active smokers, 275.0 mg/dL among never‐smokers, and 258.0 mg/dL among secondhand smokers (*p* = 0.456).

Overall, 12 participants (4.8%) had elevated fibrinogen values (> 400 mg/dL), including 10 active smokers (8.0%), 2 never‐smokers (3.2%), and no secondhand smokers, although this difference did not reach statistical significance (*p* = 0.064).

## 4 Discussion

There is an unmet need to investigate CVD risk using a validated stratification tool and blood biomarkers in Jordan. Our study assessed the 10‐year CVD risk in an apparently healthy Jordanian sample using the QRISK3 tool, alongside analysis of blood biomarker levels. The median QRISK3 score in the study population was 3.6% (range: 0.10%–80.0%), with 31.1% of participants classified as having moderate‐to‐high CVD risk (QRISK3 score ≥ 10%) and 14.3% classified as having high CVD risk (QRISK3 score > 20%). These findings indicate that a substantial proportion of this Jordanian sample faces an elevated risk of developing CVD within the next decade. Compared to the original QRISK3 validation study in the UK, our sample shows a significantly higher proportion of individuals with elevated risk (31% vs. 17.2% for ≥ 10% QRISK3 score; and 14% vs. 8.0% for > 20% QRISK3 score) [13]. According to NICE guidelines, individuals with a ≥ 10% 10‐year CVD risk should adopt lifestyle modifications such as smoking cessation, improved diet, and increased physical activity, and consider discussing cholesterol‐lowering therapies with healthcare providers, whereas those with > 20% risk require urgent management, including pharmacological intervention [14]. These findings highlight important implications for national strategies in Jordan focused on primary and secondary CVD prevention.

Smokers were found to have a higher 10‐year CVD risk compared to secondhand and never‐smokers. The median 10‐year CVD risk was highest in active smokers, followed by secondhand smokers, and lowest in never‐smokers. These findings align with existing evidence that smoking is a major risk factor for CVD, significantly increasing the likelihood of cardiovascular events [7]. Again, these findings could strengthen national efforts to control tobacco smoking, including secondhand exposure in Jordan.

Smoking is also linked to an increased incidence of atrial fibrillation, with active smokers facing more than twice the risk of developing atrial fibrillation [15]. In our study, atrial fibrillation was significantly more prevalent among active and secondhand smokers compared to never‐smokers, emphasizing the potential link between smoking and this arrhythmic condition [15]. It should be noted that we relied on self‐reporting of atrial fibrillation by the study participants without the requirement for further documentation, and this is therefore liable to bias.

Dyslipidemia, a well‐known CVD risk factor, is highly prevalent among Jordanians [16]. A study involving 4056 Jordanians reported that 44.3% had hypercholesterolemia, 41.9% had hypertriglyceridemia, 75.9% had elevated LDL levels, and 59.5% had low HDL levels [16]. Similarly, in our study, a significant proportion of participants exhibited non‐optimal lipid profile levels across all lipid parameters.

Smoking is a major risk factor for dyslipidemia [17, 18]. However, contrary to expectations, we found no statistically significant differences in the proportions of participants with non‐optimal lipid profiles (cholesterol, HDL, LDL, triglycerides, non‐HDL, LDL/HDL ratio, and cholesterol/HDL ratio) across active smokers, never‐smokers, and secondhand smokers. Previous research has shown that both the duration and intensity of smoking exacerbate lipid abnormalities. For example, individuals who smoked 1–10 cigarettes per day had a 1.74‐fold higher risk of having abnormal HDL levels compared to non‐smokers; for those smoking 10–20 cigarettes per day, the risk rose to 2.62‐fold, and for those smoking more than 20 cigarettes per day, the risk was 2.57‐fold higher than that of non‐smokers [18]. In our study, no statistically significant differences were found in the proportions of smokers with non‐optimal lipid profile parameters across different smoking intensities. However, smokers with a smoking history exceeding 10 years had lower HDL levels compared to those who had smoked for 10 years or less.

Elevated fibrinogen levels correlate with inflammation, thrombosis, and increased CVD risk [19, 20]. Smoking raises fibrinogen concentrations, thereby increasing the risk for CVD and other thrombotic events [20]. A previous study in Jordan has shown that both waterpipe smoking and cigarette smoking increased plasma fibrinogen levels in male smokers, with waterpipe smoking having a more pronounced effect (*p* < 0.01) [21]. Our study found that among all participants, 12 individuals (4.8%) had abnormal fibrinogen levels. Specifically, 8% of active smokers and 3.2% of never‐smokers had high fibrinogen levels, while none of the secondhand smokers exhibited high fibrinogen levels. Furthermore, active smokers and never smokers had higher median fibrinogen levels (268.0 mg/dl and 275.0 mg/dl, respectively) compared to secondhand smokers (258.0 mg/dl), but the difference was not statistically significant.

This study has several limitations. We used convenience sampling from two areas (Amman and Madaba) and included a relatively small sample size, which may limit generalizability to the broader Jordanian population. The lack of formal sample size calculation and potential selection bias must be acknowledged. Additionally, reliance on self‐reported smoking status and medical history may have introduced reporting inaccuracies.

In conclusion, this study demonstrates a considerable CVD risk burden within our Jordanian sample, particularly among smokers, who present a substantial proportion of the Jordanian population, as evidenced by elevated QRISK3 scores and dyslipidemia. These findings highlight the urgent need to implement routine CVD risk screening in primary healthcare settings. Implementation of integrated screening protocols incorporating QRISK3 assessment, comprehensive lipid profiling, and fibrinogen testing could facilitate early identification of high‐risk individuals and enable timely preventive interventions. Our findings also emphasize the necessity of intensified smoking cessation programs and public health policies targeting both active and passive smoking in Jordan. Future research should address the limitations of this study by incorporating larger, more diverse samples to provide a more comprehensive understanding of CVD risk in the Jordanian population and to better inform national health policies and improve cardiovascular health outcomes in Jordan.

## Author Contributions

Ahmed Salem spearheaded the project, formulated the initial research concept, provided overall supervision and guidance throughout the study, secured funding, and critically revised the manuscript. Fanar Alsmarat took part in designing the study, supported the data analysis, and helped interpret the findings. The first draft of the manuscript was written by Ahmed Salem and Fanar Alsmarat. Tasneem Al‐Awaideh, Layth Al‐Ramahi, and Yousef Shawawrah were responsible for participant recruitment and the collection of primary data. In addition, they contributed to revising the manuscript for important intellectual content. All co‐authors have thoroughly reviewed the manuscript and have provided their approval for its final version and agree to be accountable for all aspects of the work.

## Ethics Statement

Ethical approval was obtained from the Institutional Review Board committee at Hashemite University (Approval No. 33/5/2022/2023). Written informed consent was obtained from all eligible participants who met the study's inclusion criteria and agreed to participate.

## Conflicts of Interest

The authors declare no conflicts of interest.

## Transparency Statement

The lead author, Ahmed Salem, affirms that this manuscript is an honest, accurate, and transparent account of the study being reported; that no important aspects of the study have been omitted; and that any discrepancies from the study as planned (and, if relevant, registered) have been explained.

## Supporting information

Supporting File:

## Data Availability

The data supporting this research are available from the authors upon reasonable request.
