# Supplementary material for: Cardiovascular Disease Risk Estimation Using Blood Biomarkers and the QRISK3 Stratification Tool: A Cross‐Sectional Study in Jordan
Source: Health Sci Rep. 2026 Jun 4;9(6):e72388. doi: 10.1002/hsr2.72388 (PMC13238667; doi:10.1002/hsr2.72388)
Supplement: Supplementary file 1 — Supporting File: [file HSR2-9-e72388-s001.docx]

**Eligibility Screening**

1. What is your age? _______ (Must be between 25 and 64 years)
2. Are you of Jordanian Arab nationality?

- Yes
- No

1. Have you ever been diagnosed with cancer?

- Yes
- No

1. Have you ever been diagnosed with any cardiovascular disease (e.g., heart attack, stroke, angina)?

- Yes
- No

**Smoking Status Assessment**

1. What is your current smoking status?

- Active smoker (everyday smokers): has smoked at least 100 cigarettes in their lifetime and currently smokes daily.
- Secondhand smoker: has never smoked or smoked fewer than 100 cigarettes in their lifetime but regularly exposed to environmental tobacco smoke (passive smoking) by living with a smoker or working in an environment where smoking is permitted or regularly occurs indoors, with exposure of at least six hours per day.
- Never smoker: has never smoked or smoked fewer than 100 cigarettes in their lifetime and not regularly exposed to environmental tobacco smoke (passive smoking) at home or work.

1. If you are an active smoker, how many cigarettes do you usually smoke per day?

- Light smokers (≤10 cigarettes/day)
- Moderate smokers (11–19 cigarettes/day)
- Heavy smokers (≥20 cigarettes/day).

1. If you are an active smoker, for how many years have you been smoking? _______ Years

**QRISK3 Cardiovascular Risk Factors**

1. What is your sex?

- Male
- Female

1. Do you have a diagnosis of diabetes mellitus?

- Type 1
- Type 2
- No

1. Do you have a family history of cardiovascular disease (angina or heart attack in a first-degree relative under 60 years of age)?

- Yes
- No

1. Do you have chronic kidney disease (stage 3 to 5)?

- Yes
- No

1. Have you ever been diagnosed with atrial fibrillation?

- Yes
- No

1. Are you currently on treatment for high blood pressure?

- Yes
- No

1. Do you have migraine?

- Yes
- No

1. Have you been diagnosed with rheumatoid arthritis?

- Yes
- No

1. Have you been diagnosed with systemic lupus erythematosus?

- Yes
- No

1. Do you have any severe mental illness (schizophrenia, bipolar disorder, or moderate/severe depression)?

- Yes
- No

1. Are you currently taking atypical antipsychotic medications?

- Yes
- No

1. Are you currently on regular steroid tablets?

- Yes
- No
